# Fatigue and health-related quality of life in relapsing-remitting multiple sclerosis after 2 years glatiramer acetate treatment are predicted by changes at 6 months: an observational multi-center study

**DOI:** 10.1007/s00415-014-7363-2

**Published:** 2014-05-03

**Authors:** Peter Joseph Jongen, Dirk Lehnick, Jan Koeman, Stephan Frequin, Dorothea Heersema, Bert Kornips, Angelique Schyns-Soeterboek, Leo H. Visser, Paul Schiphof, Anton Valkenburg, Johan Hiel, The FOCUS Extension Study Group

**Affiliations:** 1 MS4 Research Institute, Ubbergseweg 34, 6522 KJ Nijmegen, The Netherlands; 2 Nuvisan GmbH, Wegenerstraße 13, 89231 Neu-Ulm, Germany; 3 Admiraal de Ruyter Hospital, PO Box 3200, 4380 DD Vlissingen, The Netherlands; 4 St. Antonius Hospital, Koekoekslaan 1, 3435 CM Nieuwegein, The Netherlands; 5 Academic Medical Centre Groningen, Hanzeplein 1, 9700 RB Groningen, The Netherlands; 6 Viecuri Medical Centre, Merseloseweg 130, 5801 CE Venray, The Netherlands; 7 St. Laurentius Hospital, Monseigneur Driessenstraat 6, 6043 CV Roermond, The Netherlands; 8 St. Elisabeth Hospital, Hilvarenbeekseweg 60, 5022 GC Tilburg, The Netherlands; 9 Bernhoven Hospital, Nistelrodeseweg 10, 5406 PT Uden, The Netherlands; 10 St. Franciscus Hospital, Boerhaavelaan 25, 4708 AE Roosendaal, The Netherlands; 11 St. Anna Hospital, Bogardeind 2, 5664 EH Geldrop, The Netherlands

**Keywords:** Multiple sclerosis, Relapsing remitting, Glatiramer acetate, Fatigue, Quality of life

## Abstract

Observational studies of up to 12 months duration showed that glatiramer acetate (GA) treatment of relapsing-remitting multiple sclerosis may result in decreased fatigue and improves health-related quality of life (HRQoL), with no changes in disability or mood. We investigated whether in the second year of treatment these improvements are sustained, disability or mood yet improved, and 2-year changes may be predicted by changes in the first 6 or 12 months. The multi-center FOCUS-Extension study was a prospective extension of the 12-month, international, observational FOCUS study and included 67 patients (38 treatment-naïve, 29 pre-treated) of the Dutch FOCUS cohort. Fatigue, HRQoL, depression and disability were measured by the Fatigue Impact Scale (FIS), Leeds Multiple Sclerosis Quality of Life (LMSQoL) questionnaire, Beck Depression Inventory-Short Form and the Guy’s Neurological Disability Scale. A 2-year period of GA treatment was associated with −0.52 and +0.66 standard deviation changes in mean FIS and LMSQoL scores compared to baseline, whereas disability and mood remained unchanged. For FIS and LMSQoL, the Pearson correlation coefficients between 6-month changes and 2-year scores were 0.47 and 0.50, and between 12-month changes and 2-year scores 0.65 and 0.62. After 2 years GA treatment, the improvements in fatigue and HRQoL observed at 1 year are sustained, whereas disability and mood remain unchanged compared to baseline. Moreover, the levels of fatigue and HRQoL at 2 years GA treatment are predicted by the improvements at 6 months.

## Introduction

Glatiramer acetate (GA) is a first-line disease-modifying drug (DMD) for the treatment of relapsing-remitting multiple sclerosis (MS). It reduces the frequency and severity of relapses and delays permanent disability [1, 2]. Data collected in naturalistic settings suggest that GA treatment also results in decreased fatigue [3, 4], reduction in absence from work [4], improved health-related quality of life (HRQoL) [5] and cost-effectiveness [2].

The studies that reported a beneficial effect of GA on fatigue had a relatively short duration of 6 [3] or 12 months [4, 5]. In a multi-center observational study in first-time treated patients, it was demonstrated that 12 months GA treatment is associated with an improvement in fatigue symptoms and a reduction in absence from work [4]. In an international study, we observed improvements in HRQoL and fatigue after 12 months GA treatment in persons with MS (PwMS) without prior immunomodulation or immunosuppression, with no changes in depression or disability; in pre-treated PwMS fatigue or HRQoL did neither improve nor worsen [5].

In recent years, more DMDs have become available for the treatment of RRMS, and the choices neurologists and patients have to make regarding initiation or prolongation of a treatment option have become increasingly difficult. Only a personalized and strategic use of the available DMDs will ensure that their therapeutic potential is being realized in real life. In this context, it has become more and more important to be able to predict whether an initial treatment response is sustained at prolonged treatment [6]. Given the major psychological and social impact of fatigue in PwMS improvement of fatigue may be a relevant outcome from the perspective of PwMS. It therefore seems important to know whether the improved fatigue observed at 12 months GA treatment is sustained at treatment continuation. Moreover, HRQoL is an overall subjective measure of a treatment’s effectiveness. Thus, the information that GA-induced short-term improvements in fatigue and HRQoL may predict longer term improvements would help the decision whether to continue GA treatment or not, and also promote future adherence to this therapy.

In view of these considerations, we decided to assess whether the beneficial changes in fatigue and HRQoL seen in the first year of GA treatment are continued in the second year, and to what degree 2-year levels are predicted by changes in the first 6 or 12 months. We also evaluated whether improvements in disability or mood, absent after 12 months treatment, were seen at 2 years. To this end, we extended the international 12-month observational FOCUS study in the Dutch cohort of patients for another 12 months. Here, we report the results of this FOCUS-Extension study.

## Methods

### Study design and populations

The FOCUS-Extension study was a prospective, observational, multi-center study in the Netherlands. Patients were recruited in the out-patient departments of the general hospitals, academic hospitals and MS centers that had participated in the FOCUS study [5]. The protocol was submitted to the Independent Review Board, an approved ethical committee residing in Amsterdam, the Netherlands. The Independent Review Board concluded that because of the observational design of the study a formal review by an Ethical Committee was not required; the study not meeting the criteria stated in the Dutch Medical Research Involving Human Subjects Act of 1999 [7]. Patients signed an informed consent form before any study-related procedure was performed. The study was carried out in compliance with the Helsinki Declaration. The study was funded by TEVA Pharma Netherlands and sanofi aventis Netherlands.

The inclusion criteria of the FOCUS study [5] were: (1) RRMS, (2) 18 years or older, (3) Expanded Disability Status Scale (EDSS) score <5.5, and (4) relapse- and steroid-free for at least 30 days. The exclusion criteria were: (1) hypersensitivity to GA or mannitol (2) previous treatment with GA, and (3) pregnancy or lactation. The eligibility criteria of the present FOCUS-Extension study were: (1) having participated in the FOCUS study (2) having completed month 12 assessment (3) being on GA treatment, and (4) no pregnancy or lactation.

### Outcome measures and assessment schedule

The outcome measures and assessment intervals were similar to those in the FOCUS study. Fatigue, HRQoL, depression and disability were measured by the Fatigue Impact Scale (FIS), Leeds Multiple Sclerosis Quality of Life (LMSQoL) questionnaire, Beck Depression Inventory-Short Form (BDI), and the Guy’s Neurological Disability Scale (GNDS) at 6-month intervals, i.e., at 18 and 24 months after start of GA treatment. In case of a relapse, a scheduled study visit was postponed until at least 30 days after recovery. When a patient terminated GA treatment before month 24 a final assessment occurred as for the next visit (month 18 or 24).

The FIS is a validated questionnaire examining perception of fatigue over the past month and consists of a total of 40 questions in three domains: cognitive, physical and social. Answers are rated on a 5-point scale (0–4). Total FIS scores range from 0 to 160 and higher total scores indicate more experienced fatigue [8]. The LMSQoL is a self-assessment scale that consists of eight questions, examining MS-related aspects of QoL over the past month [9, 10]. Answers are rated on a 5-point scale from 0–4 and the resulting score ranges from 8 to 32, with higher scores reflecting higher levels of well-being. The BDI is a short validated questionnaire of 13 questions [11, 12]. Answers are rated on a 4-point scale (0–3). Total scores range from 0 to 39 and higher scores indicate more depressed mood. The GNDS is a validated questionnaire measuring 12 domains of disability [13]. The GNDS score ranges from 0 to 60, where higher scores indicate increased disability. The GNDS has a good correlation to the EDSS [14].

FIS, LMSQoL and BDI were completed by patients at home within 7 days before study visit or at the clinic just prior to the visit. Investigators completed the GNDS by interview during visits.

### Statistical analyses

Changes from baseline to month 18 and 24 in FIS, LMSQoL, BDI and GNDS scores were calculated for the total group and for the groups of PwMS who belonged to the original treatment-naïve and pre-treated FOCUS groups. Differences between baseline and month 18 and month 24 assessments within a group and differences between groups were tested for significance with paired and unpaired Student *t* tests. For variables showing statistically significant changes after 24 months, we assessed to which extent the 24-month value correlated with the difference between baseline and month 6, and with the difference between baseline and month 12, by calculating Spearman coefficients. Changes were expressed as standard deviation (SD) of the baseline values. Analyses were performed according to pre-defined objectives. *P* values <0.05 were considered statistically significant. In line with the exploratory nature of the study no adjustments for multiple comparisons were made.

## Results

### Patients

In the FOCUS study 20 centers in the Netherlands recruited 142 PwMS: 73 treatment-naïve and 69 pre-treated ones. Fifteen centers participated in the FOCUS-Extension study and enrolled 67 patients, 38 originating from the FOCUS treatment-naïve group (‘naïve’ FOCUS-Extension group or ‘naïve group’) and 29 from the FOCUS pre-treated group (‘pre-treated’ FOCUS-Extension group or ‘pre-treated group’). For the total FOCUS-Extension group (*N* = 67) mean (SD) age at FOCUS study entry was 39.3 (9.7) years, and mean (SD) disease duration 5.2 (5.5) years. For the naïve group the corresponding values for age and disease duration were 38.7 (10.6) years and 3.0 (4.4) years, and for the pre-treated group 40.0 (8.5) years and 8.1 (5.4) years, respectively. The demographics of the treatment-naïve FOCUS-Extension group were very similar to those of the FOCUS treatment-naïve group [age 38.5 (9.9) years, disease duration 2.7 (4.4) years], whereas the pre-treated patients of the FOCUS-Extension study were slightly older and with a longer disease duration as compared to the FOCUS pre-treated population [age 38.8 (9.2) years, disease duration of 6.3 (5.4) years].

Month 18 and month 24 assessments were completed by 52 (77.6 %) of the patients, 29 (76.3 %) naïve and 23 (79.3 %) pre-treated.

### Comparative analyses

Mean (SD) FIS values at baseline and changes at 6, 12, 18 and 24 months GA treatment in the total, treatment-naïve and pre-treated groups are presented in Table 1. For the total extension group, as well as for the naïve and pre-treated groups, the FIS values at 18 and 24 months treatment were significantly lower than at baseline.

**Table 1:** Absolute [mean (SD), median] FIS values at baseline and absolute [mean (SD), median] and relative (SD baseline) differences from baseline at 6, 12, 18 and 24 months GA treatment in the total, treatment-naïve and pre-treated patient groups

	Total (*N* = 67)	Naïve (*N* = 38)	Pre-treated (*N* = 29)
	Mean (SD) Median ∆ (SD baseline)	Mean (SD) Median ∆ (SD baseline)	Mean (SD) Median ∆ (SD baseline)
Baseline	61.96 (31.04) 63.0 (*N* = 64)	63.42 (30.55) 62.0 (*N* = 35)	60.03 (32.93) 67.0 (*N* = 29)
Month 6	−15.63 (26.14) −12.0 −0.50 SD (*N* = 64)	−13.24 (27.54) −11.5 −0.43 SD (*N* = 35)	−18.76 (24.28) −12.0 −0.57 SD (*N* = 29)
Month 12	−10.99 (30.83) −13.0 −0.35 SD (*N* = 64)	−10.37 (31.47) −10.0 −0.34 SD (*N* = 35)	−11.79 (30.51) −14.0 −0.36 SD (*N* = 29)
Month 18	−15.27 (30.69) −15.5 (*P* = 0.0007) −0.49 SD (*N* = 52)	−13.00 (31.92) −12.5 (*P* = 0.0336) −0.43 SD (*N* = 30)	−18.36 (29.38) −19.5 (*P* = 0.0080) −0.56 SD (*N* = 22)
Month 24	−16.02 (27.45) −15.0 (*P* = 0.0001) −0.52 SD (*N* = 52)	−17.55 (26.27) −15.0 (*P* = 0.0012) −0.57 SD (*N* = 29)	−14.09 (29.35) −15.0 (*P* = 0.0312) −0.43 SD (*N* = 23)

*SD* standard deviation, ∆ difference, *B* baseline, *M6* month 6, *M12* month 12, *M18* month 18, *M24* month 24

Mean (SD) LMSQoL values at baseline and changes at 6, 12, 18 and 24 months are presented in Table 2. In the total and pre-treated patient groups the LMSQoL values at 18 months treatment were significantly higher than at baseline, but not in the naïve patients, whereas at 24 months treatment the LMSQoL values were significantly higher in all the three groups.

**Table 2:** Absolute [mean (SD), median] LMSQoL values at baseline and absolute [mean (SD), median] and relative (SD baseline) differences from baseline at 6, 12, 18 and 24 months GA treatment in the total, treatment-naïve and pre-treated patient groups

	Total (*N* = 67)	Naïve (*N* = 38)	Pre-treated (*N* = 29)
	Mean (SD) Median ∆ (SD Baseline)	Mean (SD) Median ∆ (SD Baseline)	Mean (SD) Median ∆ (SD Baseline)
Baseline	19.3 (3.69) 19.0 (*N* = 64)	19.3 (3.97) 19.0 (*N* = 35)	19.4 (3.39) 19.0 (*N* = 29)
Month 6	+1.6 (3.60) +1.0 +0.43 SD (*N* = 64)	+1.5 (3.55) +1.0 +0.38 SD (*N* = 35)	+1.62 (3.73) +1.0 +0.47 SD (*N* = 29)
Month 12	+1.8 (4.12) +2.0 +0.49 SD (*N* = 64)	+2.0 (4.48) +2.0 +0.51 SD (*N* = 35)	+1.6 (3.70) +2.0 +0.47 SD (*N* = 29)
Month 18	+1.5 (4.47) +1.0 (*P* = 0.0192) +0.41 SD (*N* = 52)	+0.9 (4.33) +1.0 (*P* = 0.2638) +0.23 (*N* = 30)	+2.32 (4.64) +2.5 (*P* = 0.0291) +0.68 (*N* = 22)
Month 24	+2.5 (4.43) +3.0 (*P* = 0.0002) +0.68 SD (*N* = 51)	+3.0 (4.79) +3.0 (*P* = 0.0024) +0.76 SD (*N* = 29)	+1.8 (3.91) +1.5 (*P* = 0.0457) +0.53 SD (*N* = 22)

*SD* standard deviation, ∆ difference, *B* baseline, *M6* month 6, *M12* month 12, *M18* month 18, *M24* month 24

Mean (SD) BDI scores at baseline and changes at 6, 12, 18 and 24 months treatment are shown in Table 3. Neither in the total group, nor in the naïve or pre-treated patients, did the values at 18 months significantly differ from baseline (*P* = 0.934, *P* = 0.3477, and *P* = 0.128, respectively). Likewise, the BDI scores at 24 months did not differ from baseline for all the three groups (*P* = 0.077, *P* = 0.345, and *P* = 0.113, respectively).

**Table 3:** Absolute [mean (SD), median] BDI values at baseline and absolute [mean (SD), median] differences from baseline at 6, 12, 18 and 24 months GA treatment in the total, treatment-naïve and pre-treated patient groups

	Total (*N* = 67)	Naïve (*N* = 38)	Pre-treated (*N* = 29)
	Mean (SD) median	Mean (SD) median	Mean (SD) median
B	6.84 (3.96) 6.0 (*N* = 64)	6.84 (4.03) 5.5 (*N* = 35)	6.83 (3.94) 6.0 (*N* = 29)
Δ B to M6	−0.18 (3.80) −1.0 (*N* = 64)	0.00 (3.36) −1.0 (*N* = 35)	−0.41 (4.36) 0.0 (*N* = 29)
Δ B to M12	−0.01 (4.01) 0.0 (*N* = 64)	0.05 (4.29) 0.0 (*N* = 35)	−0.10 (3.69) 0.0 (*N* = 29)
Δ B to M18	−0.06 (4.98) −0.5 (*N* = 52)	0.93 (5.35) 0.0 (*N* = 30)	−1.41 (4.17) −1.5 (*N* = 22)
Δ B to M24	−0.88 (3.54) −0.5 (*N* = 52)	−0.66 (3.68) 0.0 (*N* = 29)	−1.17 (3.40) −1.0 (*N* = 23)

All *P* values for comparisons between follow up and baseline >0.05

*SD* standard deviation, ∆ difference, *B* baseline, *M6* month 6, *M12* month 12, *M18* month 18, *M24* month 24

As with the BDI findings, the GNDS scores were not significantly changed in the total, naïve or pre-treated groups at 18 months (*P* = 0.180, *P* = 0.250, and *P* = 0.511, respectively), nor at 24 months (*P* = 0.061, *P* = 0.239, and *P* = 0.102, respectively) (Table 4).

**Table 4:** Absolute [mean (SD), median] GNDS values at baseline and absolute [mean (SD), median] differences from baseline at 6, 12, 18 and 24 months GA treatment in the total, treatment-naïve and pre-treated patient groups

	Total (*N* = 67)	Naïve (*N* = 38)	Pre-treated (*N* = 29)
	Mean (SD) median	Mean (SD) median	Mean (SD) median
B	9.83 (4.75) 9.5 (*N* = 64)	9.23 (4.83) 8.0 (*N* = 35)	10.55 (4.63) 10.0 (*N* = 29)
Δ B to M6	−1.05 (3.24) −0.5 (*N* = 64)	−1.49 (3.75) −1.0 (*N* = 35)	−0.52 (2.46) 0.0 (*N* = 29)
Δ B to M12	−0.50 (4.44) 0.0 (*N* = 64)	−0.29 (4.80) 0.0 (*N* = 35)	−0.76 (4.04) −2.0 (*N* = 29)
Δ B to M18	−0.94 (4.95) 0.0 (*N* = 51)	−1.24 (5.70) 0.0 (*N* = 29)	−0.55 (3.83) −0.5 (*N* = 23)
Δ B to M24	−1.43 (5.33) −1.0 (*N* = 51)	−1.43 (6.28) −1.0 (*N* = 28)	−1.43 (4.03) −1.0 (*N* = 22)

All *P* values for comparisons between follow up and baseline >0.05

SD standard deviation, ∆ difference, *B* baseline, *M6* month 6, *M12* month 12, *M18* month 18, *M24* month 24

**Fig. 1:**
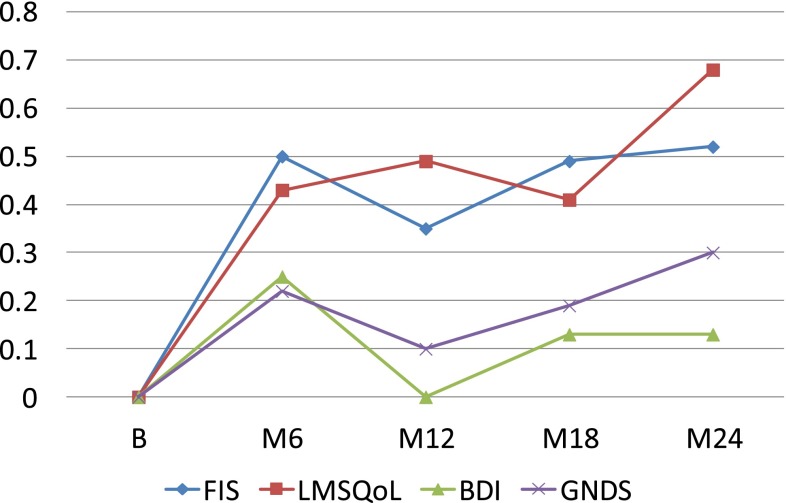
Changes in FIS, LMSQoL, BDI and GNDS scores expressed as SD baseline in RRMS patients during 24 months GA treatment. *Positive change* indicates improvement, *negative change* indicates worsening

**Fig. 2:**
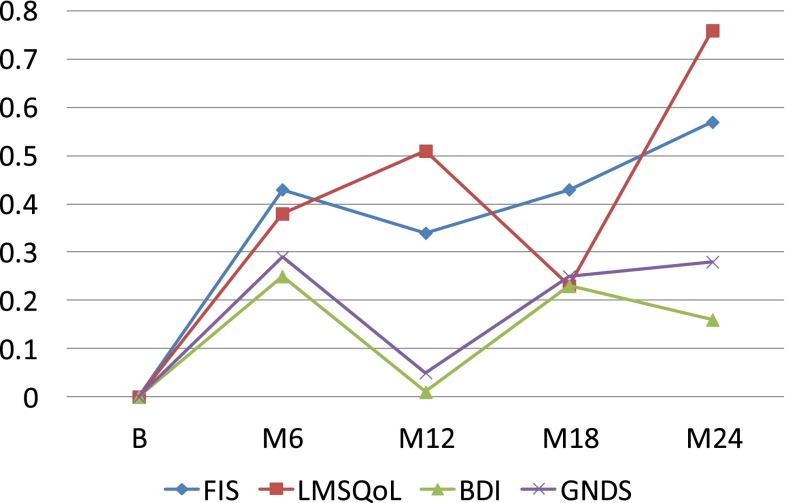
Changes in FIS, LMSQoL, BDI and GNDS scores expressed as SD baseline in treatment-naïve RRMS patients during 24 months GA treatment. *Positive change* indicates improvement, *negative change* indicates worsening

Differences in FIS, LMSQoL, BDI and GNDS scores between the naïve and the pretreated patient groups at 18 and 24 months were not significant (FIS: *P* = 0.539, *P* = 0.656; LMSQoL: *P* = 0.263, *P* = 0.346; BDI: *P* = 0.094, *P* = 0.604; GNDS: *P* = 0.624, *P* = 0.997). Mean changes in FIS, LMSQoL, BDI and GNDS scores, expressed as SD baseline, in the total group and in the treatment-naïve and pre-treated groups are shown in Figs. 1, 2 3, respectively.

**Fig. 3:**
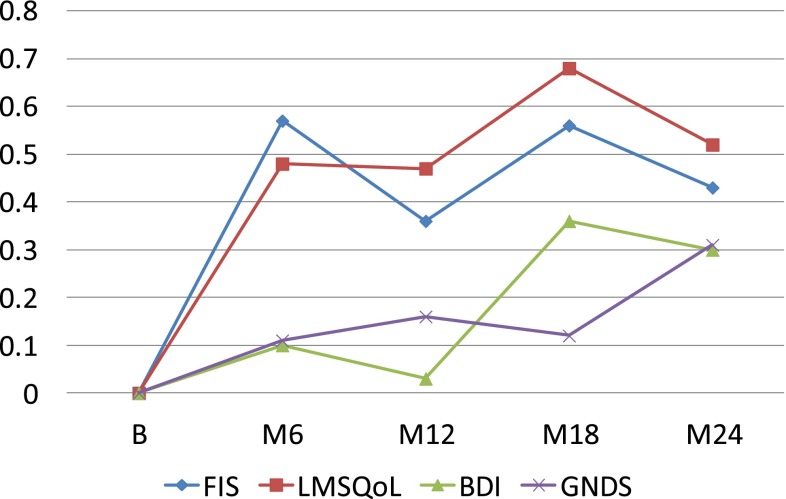
Changes in FIS, LMSQoL, BDI and GNDS scores expressed as SD baseline in pre-treated RRMS patients during 24 months GA treatment. *Positive change* indicates improvement, *negative change* indicates worsening

### Correlative analyses

The Spearman coefficients for the correlation between 24-month value and the difference between baseline to month 6, and for the correlation between 24-month value and the difference between baseline and month 12 in the total group for FIS and LMSQoL are presented in Table 5. For both measures the correlations between the initial positive changes and the absolute scores after 2 years treatment were highly significant (Table 5).

**Table 5:** Spearman coefficients for the correlations between 24-month values and the difference from baseline to month 6, and the difference from baseline to month 12 for FIS and LMSQoL in the total group

	FIS month 24 (*N* = 52)	LMSQoL month 24 (*N* = 51)
FIS Δ month 6 to baseline	0.47*	–
FIS Δ month 12 to baseline	0.65***	–
LMSQoL Δ month 6 to baseline	–	0.50**
LMSQoL Δ month 12 to baseline	–	0.62***

* *P* = 0.0005; ** *P* = 0.0002; *** *P* < 0.0001

## Discussion

In this study we observed, first, that persons with RRMS who were treated for 2 years with GA had preserved at the end of the 2-year period the improvements in fatigue and HRQoL experienced in the first year; second, that the improvements in the first 6 or 12 months correlated significantly with the 2-year level of fatigue and HRQoL; and, third, that throughout 2 years GA treatment significant changes in disability or mood were absent.

In a previous paper, we reported that in treatment-naïve RRMS, but not in pre-treated RRMS, fatigue and HRQoL significantly improved within 6 months after start of GA treatment and that the improvements were sustained at 12 months (FOCUS study) [5]. In contrast, in the present FOCUS-Extension study population improvements at 6 and 12 months were also seen in the pre-treated group, and also in the second year the levels of fatigue and HRQoL did not differ between the two groups (Figs. 1, 2, 3). As an explanation for these discrepancies we thought it likely that the pre-treated FOCUS-Extension group might not represent the original pre-treated FOCUS group. Indeed, the pre-treated persons participating in the extension study seemed to constitute a positive selection, as they had an almost fourfold (3.85) stronger decrease in the 12-month FIS score than the pre-treated FOCUS group (mean −11.79 vs. −3.06), and a fourfold greater improvement in the 12-month HRQoL score (mean +1.59 vs. +0.40). The non-representative character of the pre-treated extension group is confirmed by differences in disease duration (8.1 vs. 6.3 years) and female-to-male ratio (1.64 vs. 3.33). The fact that pre-treated extension patients had more decrease in fatigue and increase in HRQoL in the first year of GA treatment, compared to the total pre-treated group, suggests that these beneficial changes have positively influenced the decision to continue treatment. Thus, data suggest that mainly the responders—in terms of fatigue and HRQoL—in the pre-treated group continued treatment, and that the non-responders have discontinued GA treatment during or at the end of the 1-year FOCUS study.

To further compare the changes over time between the pre-treated and treatment-naive extension groups, we classified the HRQoL status as ‘improved’ (LMSQoL increase at least 3 points), ‘worsened’ (LMSQoL decrease at least -3 points) or ‘unchanged’, similar to the categories applied in the main study. It appeared that, overall, in both groups the HRQol status that had been observed at month 6 was maintained or had positively changed at month 24. E.g., in the pre-treated group five of 13 persons with ‘no change’ status at month 6 reached improvement at month 24, and in the treatment-naive group nine of 17 persons with ‘no change’ at month 6 reached ‘improved’ status at month 24. Similar trends were seen for fatigue and for the comparisons between other time points (month 12 vs. month 24, month 6 vs. month 18, month 12 vs. month 18). These observations underline the similarities between the two extension groups, and contrast with the differences found between the pre-treated and treatment-naive groups in the main study.

We choose to express the relative changes as SD of baseline values. In the whole group the mean changes after 24 months treatment were 0.52 SD for fatigue and 0.68 SD for HRQoL. In a systematic review of the literature on the interpretation of changes in HRQoL Norman et al. found that in all studies (*n* = 6) the estimated minimally important differences were close to one half SD (mean = 0.495, SD = 0.155) [15]. The frequent use of half a SD as a threshold for clinical relevance suggests that in our patient group the observed changes were indeed beneficial and deserve to be qualified as improvements.

It is not known whether the improvements in fatigue during GA treatment relate to the immunomodulation as such or to specific properties of GA. Circumstantial evidence suggests that in RRMS immunomodulation per se is capable of reducing or stabilizing fatigue. A single-center study in 50 persons with RRMS showed a decreased mean MFIS score after 12 months treatment with subcutaneously administered (sc.) interferon-beta-1a (INF-beta-1a) [16]. Moreover, in a group of 331 persons with RRMS treated for 3 years with sc. INF-beta-1a the pre-existent low mean MFIS score remained stable throughout the study period [17].

In RRMS an increase in disability results from relapses or transition to secondary progression (SP). The absence of an increase in disability during 2 years GA treatment indicates a substantial decline in the frequency and severity of relapses. In the 1-year FOCUS study, the mean (SD) annualized relapse rate decreased moderately by 27 % from 1.15 (0.62) to 0.84 (1.09); in the extension study relapses were not documented. Importantly, a report on a retrospective, observational study in MS patients who were treated with GA in clinical practice showed that during the first year of treatment the relapse rate decreased by 60 % [18]. Recently, Ford et al. published a 15-year follow-up to the pivotal phase III GA trial and concluded that MS patients who received GA for up to 15 years had reduced relapse rates, decreased disability progression, and fewer transitions to SPMS [19, 20]. Our findings of unchanged disability in RRMS patients treated for 2 years are in line with both the extension phase III and observational naturalistic data.

We found rather strong and highly significant correlations between the fatigue and HRQoL improvements in the first 6 months and the levels at 24 months. Of course, correlations between short- and long-term changes within the same parameter are to be expected. Yet, the course of fatigue and HRQoL over 24 months (Figs. 1, 2, 3) suggests that the high correlations can be explained by two phenomena: the improvements in the first 6 months and their stabilization thereafter up to month 24. Therefore, we think that beneficial changes in fatigue and HRQoL in the first 6 months of GA treatment are to a certain extent predictive of sustained long-term improvements. This is to say for treatment-naive patients, and less so for pre-treated ones, as pre-treated FOCUS-Extension patients were not representative of the pre-treated FOCUS group.

Our study was practice based and motivated by questions that may be asked in real life by PwMS who have been treated for 12 months with GA, like ‘Does my improved fatigue lasts when I continue treatment for another 12 months?’, ‘What are my chances of being still on treatment in another 12 months?’, and ‘What will be my condition at the end the second year of treatment?’. For them it may be helpful to know that three out of four persons (77.6 %) who continue treatment are still on treatment at the end of the second year, and that the levels of neurological (dis)abilities, fatigue and HRQoL that they experienced at 6 or 12 months after start of treatment, are likely to be sustained in the second year.

Finally, the documentation of HRQoL changes during DMD treatment may have practical implications. It has been demonstrated that low mental HRQoL and low self-rated health are correlated with increased disability scores 1 year later [21]. As to our study, it may be expected that patients with sustained HRQoL improvement in the second year of GA treatment have increased odds of an unchanged disability in the following year.

In conclusion, in persons with RRMS who completed 2 years GA treatment, we observed that the improvements in fatigue and HRQoL seen after 1 year treatment were sustained, and that the 2-year levels of fatigue and HRQoL correlated strongly with the improvements at 6 and 12 months. Perhaps equally important, disability and mood remained unchanged compared to baseline. Thus, our findings may help to answer real-life questions asked by PwMS in daily practice.
